# Genetic Dissection of Leaf Senescence in Rice

**DOI:** 10.3390/ijms18122686

**Published:** 2017-12-11

**Authors:** Yujia Leng, Guoyou Ye, Dali Zeng

**Affiliations:** 1 State Key Lab for Rice Biology, China National Rice Research Institute, Hangzhou 310006, China; yujialeng@caas.cn; 2 CAAS-IRRI Joint Laboratory for Genomics-assisted Germplasm Enhancement, Agricultural Genomics Institute in Shenzhen, Chinese Academy of Agricultural Sciences, Shenzhen 518120, China

**Keywords:** rice (*Oryza sativa* L.), leaf senescence, mutants, senescence-associated genes

## Abstract

Leaf senescence, the final stage of leaf development, is a complex and highly regulated process that involves a series of coordinated actions at the cellular, tissue, organ, and organism levels under the control of a highly regulated genetic program. In the last decade, the use of mutants with different levels of leaf senescence phenotypes has led to the cloning and functional characterizations of a few genes, which has greatly improved the understanding of genetic mechanisms underlying leaf senescence. In this review, we summarize the recent achievements in the genetic mechanisms in rice leaf senescence.

## 1. Introduction

Leaf senescence is the final stage of leaf development. The green leaves gradually turn to yellow, orange, red, and eventually brown and die. This process is accompanied by a series of changes at the cellular, tissue, organ, and organism levels [1]. As a form of programmed cell death (PCD), leaf senescence is primarily an age-dependent process; however, it can also be triggered prematurely by internal and external factors [2]. By integrating environmental and endogenous factors, leaf senescence provides the optimal fitness for plant development [1].

Leaf senescence is an active rather than passive process to death, and the main functions of leaf senescence are to (a) recycle and re-use the nutrients from senescing leaves into newly developing organs or offspring and (b) enhance the chance of plant survival to adapt to biotic/abiotic stresses [3,4,5]. For grain crops, leaf senescence affects grain yield and quality such as nutrient loss and incomplete filling, etc. [6]. Thus, studying the molecular mechanisms of leaf senescence will not only facilitate the understanding of this fundamental biological process, but may also provide a way to regulate leaf senescence for improving the agricultural traits of crop plants [1].

To date, many excellent reviews have described the molecular processes involved in leaf senescence in plants [5,6,7,8]. The molecular and genetic understanding of leaf senescence has been mainly gained through the use of the model plant *Arabidoposis*, which is a Dicot species. Genes controlling leaf senescence are termed as senescence-associated genes (SAGs), and many senescence-associated genes (SAGs) have been identified in plants [9,10]. Our knowledge on the molecular mechanisms underlying leaf senescence in monocots including the major cereals crops such as rice, maize, wheat, barley, and sorghum is still limited. However, with the development of molecular biology and genomics, much inspirational progress has been made in elucidating the molecular mechanisms of leaf senescence in rice. The leaf senescence database currently contains more than 130 SAGs experimentally identified in rice [11]. The objective of this review is to briefly summarize recent progress in this field.

## 2. Chloroplast Degradation Involved in Leaf Senescence

**Figure 1:**
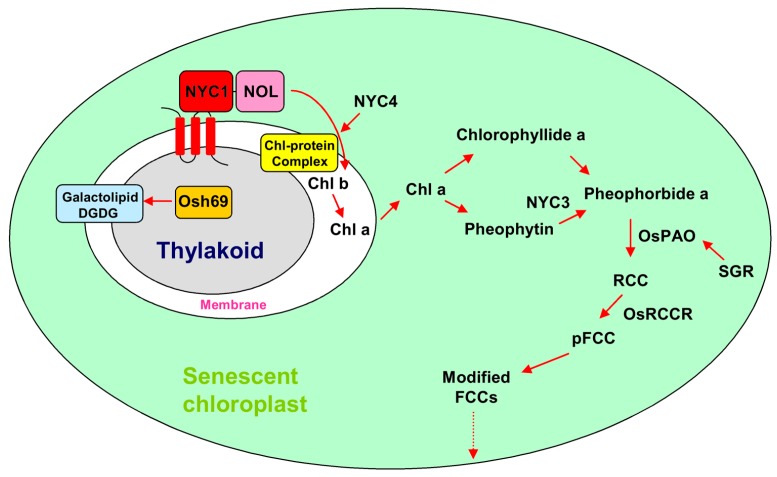
Chlorophyll degradation pathway involved in rice during leaf senescence. RCC: red chlorophyll catabolite; FCC: fluorescent chlorophyll catabolite.

During leaf senescence, chloroplasts are the first organelles to be dismantled, which can induce the production of reactive oxygen species (ROS) such as hydrogen peroxide (H_2_O_2_), superoxide anion radicals (O_2_^−^), hydroxyl radicals (OH·), and singlet oxygen (^1^O_2_). As signaling triggers, ROS influence the expression of nuclear genes, thereby causing oxidative stress and damage to the cell [12,13,14,15]. The chlorophyll (Chl) degradation pathways involved in leaf senescence have been well established in recent years [16]. Based on the current literature, several SAGs are reported to relate to chlorophyll degradation in rice by using mutations that exhibit a stay-green phenotype in the process of leaf senescence (Table 1). During the degradation of chlorophyll, the first step is the conversion from Chl b to Chl a. *NON-YELLOW COLORING 1* (*NYC1*), a chlorophyll b reductase for catalyzing the degradation chlorophyll b, encodes a chloroplast-localized short-chain dehydrogenase/reductase (SDR) and plays an important role in the degradation of the light-harvesting complex II (LHC II) and the thylakoid membrane (Figure 1) [17,18]. NYC1-LIKE (NOL), a thylakoid membrane location protein, is functionally similar to NYC1. NOL and NYC1 may form a complex to function as a chlorophyll b reductase in rice (Figure 1) [18]. Next, Chl a degradation may start with the de-chelation of Mg^2+^ by a magnesium-chelating substance which then removes phytol by pheophytinase (PPH) [19]. In rice, *NON-YELLOW COLORING 3* (*NYC3*), which encodes a plastid-localizing α/β hydrolase-fold family protein with an esterase/lipase motif, may function in removing phytol residues from pheophytin a [20]. The *STAY GREEN RICE* (*SGR*) gene encodes a senescence-inducible chloroplast stay-green protein 1. The *SGR* mutant showed chlorophyll retention, stable chlorophyll-protein complexes, and thylakoid membrane structures, but lost its photosynthetic competence during leaf senescence. Further research showed that *SGR* may be involved in regulating or participating in the activity of pheophorbide a oxygenase (*PAO*), thereby influencing the degradation of chlorophyll and pigment-protein complexes (Figure 1) [21]. *NYC4* (ortholog of *Arabidopsis THF1*) is also involved in the degradation of chlorophyll–protein complexes during leaf senescence, but its function is distinct from SGR. *NYC4* is mainly involved in the degradation of chlorophyll-protein complexes, rather than in the regulation of chlorophyll breakdown [22]. As the downstream of SGR, PAO, and red chlorophyll catabolite reductase (RCCR) are the keys in catalyzing chlorophyll degradation. Knockdown of *OsPAO* and *OsRCCR1* increased the production of ROS, resulting in leaf death and lesion mimic spots (Figure 1) [16].

The chloroplast degradation mutants above-mentioned all showed a stay-green phenotype; however, Jiao et al. [23] identified a mutant *rapid leaf senescence 1* (*rls1*), which displayed a rapid leaf senescence during chloroplast degradation. *RLS1* encodes an NB-containing protein with an ARM domain at the carboxyl terminus. The NB domain consists of three motifs and is found in many plant disease resistance proteins [23].

Galactolipids digalactosyl diacylglycerol (DGDG) and monogalactosyl diacylglycerol (MGDG) are the most abundant lipids of thylakoid membranes [24]. At the early stage of leaf senescence, the thylakoid membrane gradually breaks down, and the photosynthetic apparatus disassembles [25]. *Osh69*, a family of glycosyl hydrolases, encodes alkaline α-galactosidase. The Osh69 protein can cleave the terminal α-galactosidic bond of the galactolipid DGDG [24]. In addition, *Osh69* upregulation can be induced by many factors including darkness, hormones, and stress [24].

## 3. Phytohormones and Transcription Factors Involved in Rice Leaf Senescence

Phytohormones play vital roles in plant development including leaf senescence (Table 1) [1].

In rice, the plant hormone methyl jasmonate (MeJA) and its precursor jasmonate (JA) were the first identified senescence promoting substances [26]. *CORONATINE INSENSITIVE 1b* (*OsCOI1b*) encodes a homolog of the *Arabidopsis* jasmonate (JA) receptor COI1. The mutation of *OsCOI1b* showed methyl jasmonate (MeJA) insensitivity and delayed leaf senescence [27]. By using a metabolite-based genome-wide association study (mGWAS), Fang et al. [28] identified two major quantitative genes *OsPME1* (encoding pectin esterase) and *OsTSD2* (encoding pectin methyltransferase) that affected the content of JA. Pectin methyl esterfication is the major source of MeOH. Subsequent investigations using mutants and transgenic lines revealed an MeOH–jasmonates cascade and its epigenetic that regulates leaf senescence [28]. F-box proteins are components of E3 ubiquitin ligase with functions in a wide variety of biological processes [48]. OsFBK12, encoding an F-box protein containing a kelch repeat motif, was involved in 26S proteasome-mediated degradation by interacting with *Oryza sativa* S-PHASEKINASE-ASSOCIATED PROTEIN1-LIKE PROTEIN (OSK) and targeted the substrate S-ADENOSYL-L-METHIONINE SYNTHETASE1 (SAMS1), triggering changes in ethylene (ETH) levels for the regulation of leaf senescence [29]. *ORYZA SATIVA PREMATURE LEAF SENESCENCE* (*OsPLS1*) encoding a vacuolar H^+^-ATPase subunit A1, plays a negative regulatory role in the onset of rice leaf senescence. The *ospls1* mutant showed higher salicylic acid (SA) levels, increased ROS accumulation, and upregulation of *WRKY* genes [30]. In addition, Yamada et al. [49] found that strigolactone (SL)-deficient mutants in rice, such as *d10*, *d17*, and *d27*, showed accelerated dark-induced leaf senescence, implying that SL is involved in leaf senescence.

Several senescence-related transcription factors (TFs) are important for regulating leaf senescence (Table 1), for example, the zinc finger transcription factor *OsGATA12*, whose overexpression causes delayed leaf senescence, the reduction of leaf and tiller number, and improved rice yield. Further study showed that *OsGATA12* may be involved in decreased chlorophyll degradation [31]. Overexpression of *OsWRKY42* showed an accumulation of ROS and promoted leaf senescence by repressing *OsMT1d* expression via binding its W-box promoter in rice [32]. The class III homeodomain-leucine zipper (HD-Zip III) gene family plays important roles in plant growth and development [50]. Knockdown of an HD-Zip III member, *OsHox33*, accelerates leaf senescence in rice [33]. *ONAC106*, a senescence-associated NACs (NAM/ATAF1/ATAF2/CUC2) transcription factor, negatively regulates leaf senescence [34].

Many studies have clearly shown that transcription factors and phytohormones interactively regulate the leaf senescence process (Table 1). NACs are plant-specific transcription factors and some NACs have been confirmed to play important roles in regulating leaf senescence [51,52,53,54]. In rice, *OsNAP*/*PS1* encodes a plant-specific NAC transcriptional activator and is induced specifically by abscisic acid (ABA). Overexpression of *OsNAP*/*PS1* significantly promoted premature leaf senescence, whereas knockdown of *OsNAP*/*PS1* produced an obvious delay of leaf senescence [35]. The transcription factor SUBMERGENCE1A (SUB1A), a key regulator of submergence in rice, significantly delays dark-induced senescence by the restriction of MeJA responsiveness and ETH production [36]. A nuclear-localized zinc finger/CCCH transcription factor protein OsDOS (delay of the onset of senescence) was found to take parts of the JA pathway. Overexpression of *OsDOS* showed delayed leaf senescence, whereas knockdown caused accelerated age-dependent leaf senescence, indicating it was a negative regulator for leaf senescence [37]. In contrast, the rice *OsTZF1*, which encodes a zinc finger CCCH type family protein, is induced by many factors including ABA, JA, SA, drought, high-salt, and H_2_O_2_. Overexpression of *OsTZF1* showed delayed seed germination, growth retardation, delayed leaf senescence, improved tolerance to high-salt and drought stresses, and caused opposite phenotypes [38]. OsMYC2, a JA-inducible basic helix-loop-helix transcriptional factor, is a positive regulator of leaf senescence by the direct regulation of some SAGs in rice. Overexpression of *OsMYC2* significantly promoted leaf senescence and a reduction in chlorophyll content, and was negatively regulated by OsJAZ8 (a JA ZIM-domain protein), involved in the JA signaling pathway in rice [26]. In addition, a recent study showed that miR319-controlled TCP transcription factors were involved in regulating JA content and leaf senescence [55].

Aside from the transcription factors mentioned above, based on microarray data, Liu et al. (2016) concluded that the W-box and G-box *cis*-elements may function as positive regulators affecting rice leaf senescence (Table 1) [56].

## 4. Energy Metabolism Pathway Regulated Rice Leaf Senescence

**Figure 2:**
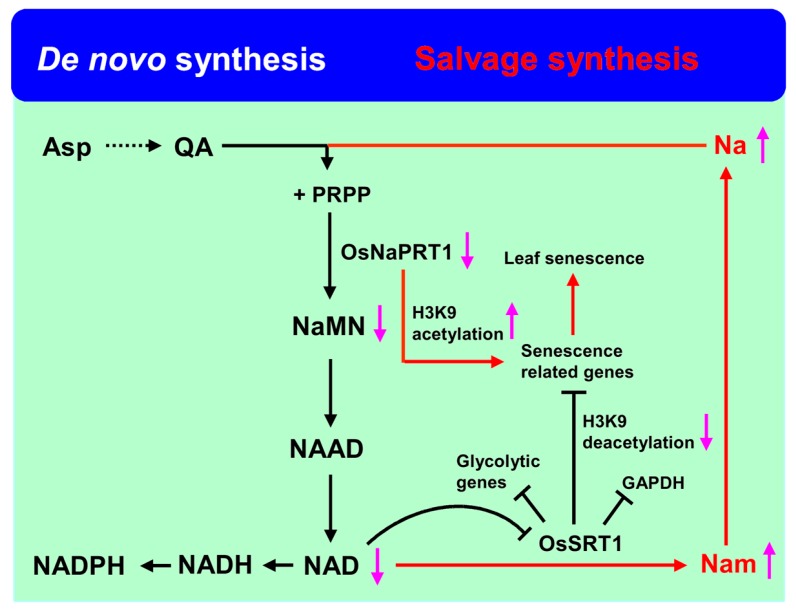
NAD synthesis and catabolic pathways involved in rice leaf senescence. Asp: aspartate, QA: quinolinic acid, Na: nicotinic acid, Nam: nicotinamide, PRPP: 5-phosphoribosyl-1-pyrophosphate, NaMN: nicotinate mononucleotide, NAAD: nicotinic acid adenine dinucleotide, NAD: nicotinamide adenine dinucleotide.

Nicotinamide adenine dinucleotide (NAD) and its derivative nicotinamide adenine dinucleo-tide phosphate (NADP) are important energy metabolite pathways involved in redox reactions in living organisms [57,58]. It was shown that NAD depletion could prevent cell death in vivo to maintain the balance of the internal environment [59]. In *Arabidopsis*, there are two NADP biosynthetic pathways: de novo and the salvage pathway [60,61]. In the salvage pathway, SIR2, an NAD^+^-dependent histone deacetylase, plays a crucial role in converting NAD to nicotinamide (Nam) [39,40]. In rice, there are two SIR2 homologous genes, *OsSRT1* (*OsSIRT701*) and *OsSRT2* (*OsSIRT702*) [62]. RNA interference of *OsSRT1* results in an increase of histone H3K9 acetylation and a decrease of H3K9 dimethylation, H_2_O_2_ accumulation, DNA fragmentation, programmed cell death, and mimicking plant lesions, and its overexpression enhances the tolerance of redox [40]. Recent research indicated that OsSRT1 could regulate carbon metabolic flux through the repression of glycolysis by the deacetylation of both histone and glycolytic glyceraldehyde-3-phosphatedehydrogenase (GAPDH) (Figure 2) [63].

Downstream of NAD, Nam from nicotinate mononucleotide (NaMN) was catalyzed by two enzymes: nicotinamidase and nicotinate phosphoribosyltransferase (NaPRTase) [59,61]. In rice, a mutation of *NaPRTase, LTS1,* revealed increased concentrations of nicotinate and nicotinamide as well as decreased NAD content. Further research indicated that the decreased NAD repressed the expression of *OsSRTs* and would result in a lower deacetylation ability of *OsSRTs*, hence activating senescence-related genes by increasing the acetylation of histone H3K9, leading to leaf senescence in rice (Figure 2) [39].

## 5. Nitrogen Remobilization Involved in Rice Leaf Senescence

Nitrogen remobilization increases nitrogen use efficiency and plays an important role in sustainable agriculture. Nitrogen molecules have a major presence in proteins and nucleic acids, and are transported in the form of amino acids (particularly glutamine and asparagine) from the senescence leaves to new parts [64]. The metabolism of glutamate and γ-aminobutyric acid (GABA) plays an important role in nitrogen circulation [65]. During glutamate metabolism, glutamine synthetase catalyzes ammonia and 2-oxoglutarate into glutamine, whereas glutamate synthase (or glutamine 2-oxoglutarate aminotransferase, GOGAT) catalyzes the reversible conversion of glutamine into glutamate [65,66]. In higher plants, GOGAT has two isoforms: Fd-GOGAT and NADH-GOGAT. Fd-GOGAT is predominantly located in the chloroplasts of photosynthetic tissues, and NADH-GOGAT is present in non-photosynthesizing cells [66]. In rice, the *gogat1* mutant exhibited chlorosis under natural conditions and less extent premature leaf senescence under low light conditions. Meanwhile, the *gogat1* mutant showed a reduced seed setting rate and increased grain protein and amino acid content. This result showed that OsFd-GOGAT plays an important role in nitrogen remobilization during leaf senescence [41].

The transferring glutamate to succinate via GABA is called the GABA metabolism or GABA shunt [65]. As a temporary storage of nitrogen, enhanced GABA can inhibit the synthesis of glutamine during senescence [67]. GABA:pyruvate-transaminase catalyzes GABA into succinic semialdehyde (SSA). SSA is then catalyzed into succinate by succinic semialdehyde dehydrogenase (SSADH) and goes into a tricarboxylic acid (TCA) cycle [65]. In rice, Osl2, encoding γ-aminobutyric acid (GABA):pyruvate transaminase, is upregulated and plays a key role in nitrogen metabolism during leaf senescence [42,65].

## 6. Other Genes Involved in Leaf Senescence

Recent research has shown that cell-wall-related genes are involved in the regulation of leaf senescence. The *DWARF AND EARLY-SENESCENCE 1* (*DEL1*) gene encodes a pectate lyase precursor. Loss of function of *DEL1* decreased total pectate lyase (PEL) activity, increased the degree of methylesterified homogalacturonan (HG), and perturbed cell wall composition and structure, resulting in triggering ROS activity, thereby leading to leaf senescence [43].

UDP-*N*-acetylglucosamine pyrophosphorylase (UAP) is widely distributed in living organisms [44]. Wang et al. [44] cloned the *SPOTTED LEAF 29* (*SPL29*) gene, which encodes UAP1 in rice. The *spl29* mutant displayed many changes involved in chloroplast degradation, chlorophyll loss and photosystem II decline, enhanced resistance to bacterial blight inoculation, increased malondialdehyde content and ROS, upregulated SAGs and defence response genes, downregulated photosynthesis-related genes, etc. [44].

Actin filament plays an important role in many endomembrane processes such as vacuole formation, endocytosis of plasma membrane (PL), and vesicle transport from the Golgi complex, etc. [68,69,70,71,72]. The ARP2/3 complex as a key regulator of actin filament nucleation can be inactive by itself and active by the SCAR/wave complex in plants [73]. The SCAR/WAVE complex is highly conserved, and deficiency in the SCAR/WAVE complex in plants often leads to morphological changes [45]. In rice, *EARLY SENESCENCE 1* (*ES1*) encodes a SCAR-LIKE PROTEIN2, which plays an important role in leaf senescence. The *es1* mutant shows a short and irregular arrangement of actin filaments. The changes to the actin filaments increase the water loss of leaves, thereby leading to leaf senescence [45].

The SWEET family plays important roles in plant growth and development. In rice, overexpression of *OsSWEET5*, a novel sugar transporter family, caused growth retardation and precocious senescence at the seedling stage [46].

Glycine decarboxylase complex (GDC) is a multi-protein complex, which plays a major role in the photorespiration of plants [74]. Under ambient CO_2_, knockdown of *OsGDCH* caused leaf senescence due to chlorophyll loss, protein degradation, chloroplast breakdown, and autophagy, as well as ROS accumulation [47].

## 7. Perspectives

Leaf senescence is a very complex phenomenon. It is an evolutionarily acquired developmental strategy to adapt to internal and external factors [2]. Leaves are the main locations of plant photosynthesis, most of the carbon in mature rice grains originates from leaf photosynthesis [75]. Timely leaf senescence can make plants accumulate enough nutrients for assimilation, while excessive leaf senescence can lead to decreased plant photosynthetic capacity and assimilation capacity, thereby reducing crop yield and quality. In addition, adequate remobilization of nutrients increases the usage efficiency of crops, thereby reducing the use of fertilizers [8].

Rice (*Oryza sativa* L.) is an important staple food that feeds more than half of the world’s population, mainly in Asia [76]. Achieving increases in rice grain-yield is a permanent topic of concern for over-increasing populations [77]. Delaying leaf senescence, particularly of the flag leaf, would help to increase grain yield [3]. The stay-green traits have been used in breeding to enhance stress resistance and increase grain yield, although the relationships between leaf senescence and crop yield and quality have not yet been well characterized [78]. Systematically elucidating the molecular mechanisms of leaf senescence will provide breeders with new tools/options for further improving many important agronomic traits in future.

As summarized in this review, significant progress has been made in the cloning and functional characterization of leaf SAGs in rice in the last few decades. However, the discussion in this review only focuses on the role of single genes in the onset of senescence. The main reason for this is based on the study progress of leaf senescence in rice. Therefore, a deeper understanding of leaf senescence will provide more insights into the improvement of crop productivity.

## Figures and Tables

**Table 1:** Leaf senescence related genes in rice.

Gene	Accession Number	Functional Annotation	Mutant Phenotype	Overexpression Phenotype	Regulatory Role **^Δ^**	Ref.
*OsPAO*	LOC_Os03g05310	Pheophorbide a oxygenase	early	unknown	−	[16]
*OsRCCR1*	LOC_Os10g25030	Red chlorophyll catabolite reductase	early	unknown	−	[16]
*NYC1*	LOC_Os01g12710	Short-chain dehydrogenase/reductase	delayed	unknown	+	[17]
*NOL*	LOC_Os03g45194	Short-chain dehydrogenase/reductase	delayed	unknown	+	[18]
*NYC3*	LOC_Os06g24730	α/β hydrolase-fold family protein	delayed	unknown	+	[20]
*SGR*	LOC_Os09g36200	Senescence-inducible chloroplast stay-green protein 1	delayed	early	+	[21]
*NYC4*	LOC_Os07g37250	THYLAKOID FORMATION 1	delayed	unknown	+	[22]
*RLS1*	LOC_Os02g10900	NB-ARC domain containing protein	early	early	−	[23]
*Osh69*	LOC_Os08g38710	Alkaline α-galactosidase	unknown	unknown	+	[24]
*OsMYC2*	LOC_Os10g42430	JA-inducible basic helix-loop-helix transcription factor	unknown	early	+	[26]
*OsCOI1b*	LOC_Os05g37690	F-box domain and LRR containing protein	delayed	unknown	+	[27]
*OsPME1*	LOC_Os01g57854	Pectinesterase	delayed	early	+	[28]
*OsTSD2*	LOC_Os02g51860	Pectin methyltransferase	delayed	unknown	+	[28]
*OsFBK12*	LOC_Os03g07530	F-box protein containing a kelch repeat motif	early	delayed	−	[29]
*OsPLS1*	LOC_Os06g45120	Vacuolar H^+^-ATPase subunit A1	early	unknown	−	[30]
*OsGATA12*	LOC_Os03g61570	GATA-like zinc finger transcription factor	unknown	delayed	−	[31]
*OsWRKY42*	LOC_Os02g26430	Nuclear transcriptional repressor	unknown	early	+	[32]
*OsHox33*	LOC_Os12g41860	Class III homeodomain-leucine zipper gene family	early	unknown	−	[33]
*ONAC106*	LOC_Os01g66120	NAC domain transcription factor	delayed	unknown	+	[34]
*OsNAP*/*PS1*	LOC_Os03g21060	No apical meristem	delayed	early	+	[35]
SUB1A *	No	Submergence tolerance regulator	unknown	delayed	−	[36]
*OsDOS*	LOC_Os01g09620	Nuclear-localized CCCH-type zinc finger protein	early	delayed	−	[37]
*OsTZF1*	LOC_Os05g10670	CCCH-tandem zinc finger protein	early	delayed	−	[38]
*LTS1/OsNaPRT1*	LOC_Os03g62110	Nicotinate phosphoribosyltransferase	early	unknown	−	[39]
*OsSRT1*	LOC_Os04g20270	NAD^+^-dependent histone deacetylases	early	delayed	−	[40]
*OsFd-GOGAT*	LOC_Os07g46460	Ferredoxin-dependent glutamate synthase	early	unknown	−	[41]
*Osl2*	LOC_Os04g52450	γ-aminobutyric acid (GABA):pyruvate transaminase	unknown	unknown	+	[42]
*DEL1*	LOC_Os10g31910	Pectate lyase precursor	early	unknown	−	[43]
*SPL29*	LOC_Os08g10600	UDP-*N*-acetylglucosamine pyrophosphorylase 1	early	unknown	−	[44]
*ES1/TUTOU1*	LOC_Os01g11040	SCAR-like protein 2	early	unknown	−	[45]
*OsSWEET5*	LOC_Os05g51090	Sugar transporter family	unknown	early	+	[46]
*OsGDCH*	LOC_Os10g37180	Glycine decarboxylase complex H subunit	early	Unknown	−	[47]

**^Δ^** + positive regulation; − negative regulation; * absent in Nipponbare and therefore without an LOC number. NB-ARC: nucleotide-binding, Apaf-1, R proteins, and Ced-4; JA: jasmonate; LRR: Leucine-rich repeat; GATA: GATA motif; NAC: NAM/ATAF1/ATAFC2; CCCH: C-x8-C-x5-C-x3-H; UDP: uridine diphosphate; SCAR: suppressor of cAMP receptor.

## Abbreviations

ABA	abscisic acid
Chl	chlorophyll
ETH	ethylene
DGDG	digalactosyl diacylglycerol
GAPDH	glyceraldehyde-3-phosphatedehydrogenase
HG	homogalacturonan
H_2_O_2_	hydrogen peroxide
JA	jasmonate
LHCⅡ	light-havesting complexⅡ
MeJA	methyl jasmonate
MGDG	monogalactosyl diacylglycerol
mGWAS	metabolite-based genome-wide association study
NAD	nicotinamide adenine dinucleotide
NADP	nicotinamide adenine dinucleotide phosphate
Nam	nicotinamide
NaMN	nicotinate mononucleotide
^1^O_2_	singlet oxygen
O_2_^−^	superoxide anion radical
OH	hydroxyl radical
PCD	programmed cell death
PEL	pectate lyase
PL	plasma membrane
PPH	pheophytinase
ROS	reactive oxygen species
SA	salicylic acid
SAGs	senescence-associated genes
SL	strigolactone
SSA	succinic semialdehyde
SSADH	succinic semialdehyde dehydrogenase
TCA	tricarboxylic acid
TFs	transcription factors
